# A Multimodal Score Accurately Classifies Fontan Failure and Late Mortality in Adult Fontan Patients

**DOI:** 10.3389/fcvm.2022.767503

**Published:** 2022-03-10

**Authors:** Peter Kramer, Anastasia Schleiger, Marie Schafstedde, Friederike Danne, Johannes Nordmeyer, Felix Berger, Stanislav Ovroutski

**Affiliations:** ^1^Department of Congenital Heart Disease/Pediatric Cardiology, German Heart Centre Berlin, Berlin, Germany; ^2^Institute for Cardiovascular Computer-Assisted Medicine, Charité - Universitätsmedizin Berlin, Berlin, Germany; ^3^Berlin Institute of Health, Berlin, Germany; ^4^Department of Pediatric Cardiology, Charité - Universitätsmedizin Berlin, Campus Virchow-Klinikum, Berlin, Germany; ^5^German Centre for Cardiovascular Research (DZHK), Partner Site Berlin, Berlin, Germany

**Keywords:** Fontan operation, univentricular heart disease, adult congenital heart disease, Fontan failure, late mortality

## Abstract

**Objectives:**

Despite the outstanding success of the Fontan operation, it is a palliative procedure and a substantial number of patients experience late failure of the Fontan circulation. Clinical presentation and hemodynamic phenotypes of Fontan failure are considerably variable. While various parameters have been identified as risk factors for late Fontan failure, a feasible score to classify Fontan failure and possibly allow timely risk stratification is lacking. Here, we explored the possibility of developing a score based on hemodynamic, clinical and laboratory parameters to classify Fontan failure and mortality.

**Methods:**

We performed a retrospective study in our cohort of adult Fontan patients from two institutions [*n* = 198, median follow-up after Fontan 20.3 (IQR 15.6–24.3) years], identifying those patients with clinical Fontan failure (*n* = 52, 26.3%). Various hemodynamic, echocardiographic, laboratory and clinical data were recorded and differences between patients with and without Fontan failure were analyzed. We composed a Fontan Failure Score containing 15 parameters associated with Fontan failure and/or mortality and assessed its accuracy to discriminate between patients with and without late Fontan failure as well as late mortality and survival.

**Results:**

Late failure occurred at a median of 18.2 (IQR 9.1–21.1) years after Fontan completion. Mortality associated with Fontan failure was substantial (25/52, 48.1%) with freedom of death/transplantation/take-down of 64% at 5 years and 36% at 10 years after onset of Fontan failure, respectively. Patients with Fontan failure had a significantly higher median Fontan Failure Score compared to non-failing Fontan patients [8 points (IQR 5–10) vs. 2 points (IQR 1-5), *p* < 0.001]. The score accurately classifies Fontan failure as well as mortality as assessed with receiver operating characteristic analysis. Area under the curve of the Fontan Failure Score was 0.963 (95% CI 0.921; 0.985, *p* < 0.001) to discriminate failure and 0.916 (95% CI 0.873; 0.959, *p* < 0.001) to classify mortality.

**Conclusion:**

We have developed an uncomplex yet remarkably accurate score to classify Fontan failure and late mortality in adult Fontan patients. Prospective validation and most likely refinement and calibration of the score in larger and preferably multi-institutional cohorts is required to assess its potential to predict the risk of Fontan failure and late mortality.

## Introduction

The Fontan operation represents a milestone in the treatment of children with complex univentricular heart disease (1, 2). The continuous improvements during the past 50 years in surgical techniques, perioperative care, preoperative selection criteria and medical as well as interventional treatment strategies have resulted in substantial decreases in early and late mortality. Thus, a growing number of these patients are entering adolescence and adulthood today (2–4). Despite this outstanding success, it is a palliative procedure and the profoundly unphysiological hemodynamic principles of the Fontan circulation are fundamentally unchanged (2, 5, 6). Unavoidably, the Fontan circulation pathophysiology results in chronic venous congestion and reduced ventricular preload with a chronic low output state. Chronic Fontan circulation failure is associated with progressive clinical heart failure and ultimately premature death or the need of cardiac transplantation (4, 7–9). Importantly, with a continuously growing number of Fontan patients entering into adulthood, a substantial increase in the incidence of Fontan failure can be expected within the near future (10).

Notably, Fontan failure is not a uniform process and the hemodynamic alterations observed in patients with a failing Fontan circulation vary considerably, which has resulted in the conceptualization of distinct hemodynamic phenotypes of Fontan failure (5, 11, 12). Given the heterogeneous and likely multifactorial causes of Fontan failure and the clear lack of evidence concerning optimal therapy of these complex patients, therapeutic success is often limited, especially in patients without surgically or interventionally addressable hemodynamic issues (13–15). Early identification of patients with developing or apparent Fontan failure might allow a more timely and targeted initiation of therapies in order to delay the most likely inevitable hemodynamic demise of the Fontan circulation. Moreover, in light of limited therapeutic options, considerable mortality and increasing transplantation risk, it seems advisable to timely evaluate patients with failing Fontan for cardiac transplantation. However, to determine the optimal time point is a difficult task given the current knowledge gaps. This emphasizes the need of a consensus on precisely defining Fontan failure as well as the necessity of a feasible means to assess varying grades of Fontan failure in terms of risk stratification (16).

A feasible clinical scoring system aiming at classifying Fontan failure severity might prove valuable to address these subjects but is, however, not available at present. Therefore, in our present study we explored the possibility of developing an uncomplex and reproducible yet comprehensive multimodal Fontan Failure Score based on hemodynamic, clinical and laboratory parameters to classify Fontan failure and late mortality.

## Methods

We performed a retrospective cohort study identifying adult Fontan patients from our institutional databases. Patients were included in the study if they were followed in our institutions (German Heart Center Berlin and Charité - Universitätsmedizin Berlin) and ≥18 years of age at their last follow-up visit during the study period from April 1996 to April 2021 (Supplementary Figure 1). Latest available demographic, clinical, echocardiographic, cardiopulmonary exercise testing, and hemodynamic data as well as laboratory parameters were recorded from medical charts. The study was approved by the institutional review board and the appropriate Institutional Ethics Committee (Decision Number EA2/126/15); requirement of individual informed consent was waived due to the retrospective nature of the study.

### Definition of Fontan Failure

Fontan failure was *a priori* defined as meeting at least one of the following clinical criteria: NYHA functional class IV, NYHA functional class III for ≥12 months without sustained improvement, >2 unscheduled hospital admissions within 12 months for heart failure symptoms, evaluation/listing for cardiac transplantation, and active protein-losing enteropathy and/or plastic bronchitis without remission for ≥6 months. Fontan failure according to our definition was classified for the last available follow-up visit. Onset of failure was then determined by recording the time point at which any criterion of Fontan failure was first met. Active protein-losing enteropathy was defined as a combination of persistent diarrhea and/or recurring edema and/or pleural effusions and/or ascites, decreased serum albumin (<3.5 g/dL) and total serum protein levels (<6.0 g/dL) and confirmation of intestinal protein loss with increased fecal alpha-1 antitrypsin levels (17). Active plastic bronchitis was defined as symptomatic episode requiring hospital admission within the last 6 months in patients with known plastic bronchitis. Initial diagnosis was required to be confirmed by observation of typical fibrinous bronchial casts (18).

### Data Acquisition

Data recorded included baseline details of cardiac anatomy and Fontan operation as well as history of arrhythmias (supraventricular and ventricular tachycardia, pacemaker-requiring bradyarrythmia) and most recent demographic data, clinical status and cardiovascular medication obtained at the last available follow-up visit. For our study, history of supraventricular tachycardia (SVT) was defined as occurrence of at least one episode of symptomatic sustained SVT requiring either initiation/adaptation of antiarrhythmic treatment, cardioversion or invasive electrophysiological study documented in patients' medical charts. History of ventricular tachycardia (VT) was defined as either sustained VT recorded during Holter monitoring and/or syncope or implantation of implantable cardioverter/defibrillator after detection of non-sustained VT by Holter monitoring, as documented in medical charts. Cardiovascular medications were grouped and recorded as presented in Table 1. A combination of loop diuretics with any other diuretic was counted as two medications. For the exception of sotalol, ß-blockers were not grouped as antiarrhythmic medications. Invasive hemodynamic data were collected from the last cardiac catheterization performed during follow-up. Cardiac catheterizations were exclusively performed under conscious sedation with spontaneous breathing according to our routine institutional protocols. In our institution, we generally do not perform routine cardiac catheterizations during follow-up at fixed ages or intervals; however, we have a policy of liberally performing clinically indicated catheterizations. Systemic ventricular end-diastolic pressures (SVEDP), mean Fontan/pulmonary artery pressure (mPAP) and pulmonary capillary wedge pressure were recorded; mean transpulmonary pressure gradient was calculated as mPAP—mean pulmonary capillary wedge pressure. Pulmonary vascular resistance (PVR) was determined by Fick's principle as previously described using oximetry (19). PVR is reported in Wood units (WU) and was indexed to body-surface area (pulmonary vascular resistance index PVRi, WU^*^m^2^) (20). Echocardiographic data from last available follow-up examinations were extracted. To assess the systolic function of the single ventricle, ejection fraction (EF) was quantified using the modified Simpson's method (21). The degree of atrioventricular valve regurgitation was classified as absent/trace, mild, moderate or severe as documented by visual or quantitative assessment (22). Peak oxygen consumption (VO_2_peak), defined as highest mean oxygen uptake throughout exercise over a 30 s period, was recorded from the most recent cardiopulmonary exercise testing (CPET) and is reported as absolute as well as percentage of age- and sex-specific reference values, respectively. Laboratory parameters collected included N-terminal pro-brain natriuretic peptide (NT-proBNP), red cell distribution width (RDW), total bilirubin, γ-glutamyltransferase (gGT), aspartate aminotransferase (AST), alanine aminotransferase (ALT), creatinine and cystatin C. Estimated glomerular filtration rate (eGFR) was calculated according to the Chronic Kidney Disease Epidemiology Collaboration (CKD-EPI) formulae based on serum creatinine or, if additionally available, creatinine and cystatin C (23, 24). In patients with reoperation mortality, only data preceding the time point of reoperation was recorded.

**Table 1:** Characteristics of adult Fontan patients.

	**Entire cohort *N* = 198**	**Adults with failing Fontan *n* = 52**	**Adults without failing Fontan *n* = 146**	***p***
**Baseline characteristics**				
Anatomy (*n*)				0.350
Tricuspid atresia	74 (37.4%)	20 (38.5%)	54 (37.0%)	
Double inlet left ventricle	36 (18.2%)	10 (19.2%)	26 (17.8%)	
Unbalanced AVSD	18 (9.1%)	6 (11.5%)	12 (8.2%)	
HLHS	6 (3.0%)	0 (0.0%)	6 (4.1%)	
Complex TGA/ccTGA	39 (19.7%)	11 (21.2%)	28 (19.2%)	
PA/IVS	10 (5.1%)	0 (0.0%)	10 (6.8%)	
Other	15 (7.6%)	5 (9.6%)	10 (6.8%)	
Predominant ventricular morphology				0.407
Left ventricle	160 (80.1%)	40 (76.9%)	120 (82.2%)	
Right ventricle	38 (19.9%)	12 (23.1%)	26 (17.8%)	
Heterotaxy (*n*)	17 (8.6%)	4 (7.7%)	13 (8.9%)	0.788
Age at Fontan (years)	4.9 (3.4–11.3)	10.0 (4.2–18.1)	4.5 (3.3–7.4)	**<0.001**
Fontan type (*n*)				**<0.001**
Extracardiac TCPC	97 (49.0%)	12 (23.1%)	85 (58.2%)	
Intracardiac TCPC	74 (37.4%)	22 (42.3%)	52 (35.6%)	
APC	21 (10.6%)	14 (26.9%)	7 (4.8%)	
AVC	6 (3.0%)	4 (7.7%)	2 (1.4%)	
**Last follow-up**				
Follow-up after Fontan (years)	20.3 (15.6–24.3)	22.7 (15.2–25.7)	19.6 (15.6–23.6)	0.081
Age at last follow-up (years)	25.9 (21.4–32.8)	31.4 (25.4–39.1)	24.1 (20.7–29.8)	**<0.001**
Mortality during follow-up (*n*)	27 (13.6%)	25 (48.1%)	2 (1.4%)	**<0.001**
Ejection fraction (%)	51 (46–58)	48 (37–55)	55 (49–59)	**<0.001**
Atrioventricular valve incompetence (*n*)				**<0.001**
None/trace	74 (37.4%)	11 (21.2%)	63 (43.2%)	
Mild	73 (36.7%)	17 (32.7%)	56 (38.4%)	
Moderate	31 (15.7%)	13 (25.0%)	18 (12.3%)	
Severe	12 (6.1%)	11 (21.2%)	1 (0.7%)	
mPAP (mmHg)	12 (10–14)	15 (11–17)	11 (9–13)	**<0.001**
SVEDP (mmHg)	8 (6–12)	10 (8–14)	8 (6–10)	**<0.001**
TPG (mmHg)	4 (3–5)	4 (3–5)	4 (3–5)	0.741
PVRi (WU*m^2^)	0.94 (0.66–1.21)	1.02 (0.84–1.44)	0.86 (0.64–1.14)	**0.018**
TCS (%)	94 (91–96)	90 (87–94)	95 (93–97)	**<0.001**
History of arrhythmia (*n*)				
Supraventricular tachycardia	68 (34.3%)	32 (61.5%)	36 (24.7%)	**<0.001**
Ventricular tachycardia	15 (7.6%)	11 (21.2%)	4 (2.7%)	**<0.001**
Pacemaker-requiring bradycardia	52 (26.3%)	25 (48.1%)	27 (18.5%)	**<0.001**
NT-proBNP (pg/mL)	187.9 (76.8–468.9)	872.8 (375.4–1930.0)	114.0 (55.1–213.1)	**<0.001**
RDW (%)	13.9 (13.1–16.3)	17.0 (14.8–18.5)	13.4 (12.8–14.3)	**<0.001**
Cystatin C (mg/L)	1.1 (1.0–1.2)	1.2 (1.1–1.6)	1.1 (0.9–1.2)	**0.008**
Creatinin (mg/dL)	0.85 (0.75–0.97)	0.90 (0.80–1.18)	0.82 (0.75–0.91)	**0.001**
eGFR (ml/min/1.73 m^2^)	100 (86–119)	86 (68–108)	105 (91–120)	**<0.001**
AST (U/L)	30 (26–30)	30 (26–38)	31 (26–36)	0.999
ALT (U/L)	32 (21–41)	22 (15–39)	33 (25–41)	**0.009**
Total bilirubin (mg/dL)	1.10 (0.82–1.70)	1.30 (0.79–2.16)	1.10 (0.82–1.50)	0.340
gGT (U/L)	76 (49–127)	96 (62–180)	68 (46–109)	**0.014**
CPET VO_2_peak (mL/kg/min)	21.7 (16.7–27.1)	14.1 (11.5–17.2)	23.6 (20.0–28–1)	**<0.001**
CPET VO_2_peak (% of reference)	58 (45–70)	38 (32–46)	64 (54–73)	**<0.001**
**Cardiovascular medication**				
Loop diuretics	61 (30.8%)	45 (86.5%)	15 (10.3%)	
Other diuretics	77 (38.9%)	43 (82.7%)	34 (23.3%)	
ß-blockers	80 (40.4%)	38 (73.1%)	42 (28.8%)	
ACE inhibitor/AT1 blocker	81 (40.9%)	20 (38.5%)	61 (41.8%)	
Pulmonary vasodilator	36 (18.2%)	19 (36.5%)	17 (11.6%)	
Sacubitril/valsartan	4 (2.0%)	3 (5.8%)	1 (0.7%)	
Antiarrhythmics	52 (26.3%)	29 (55.8%)	23 (15.8%)	
Inotropes	10 (5.1%)	10 (19.2%)	0 (0.0%)	
Any medication	152 (76.8%)	52 (100%)	100 (68.5%)	**<0.001**
>1 medications	107 (54.0%)	51 (98.1%)	56 (38.4%)	**<0.001**
>2 medications	72 (36.4%)	47 (90.4%)	25 (17.1%)	**<0.001**

*Data are presented as median (interquartile range) or frequencies (percentages). P-values < 0.05 are considered statistically significant and highlighted in bold*.

*Missing data: failing Fontan group AST n = 16 (30.8%); ALT n = 5 (9.6%); bilirubin n = 15 (28.8%); NT-proBNP n = 8 (15.4%); eGFR, gGT n = 2 (3.8%); RDW n = 3 (5.8%); cardiopulmonary exercise testing VO2peak n = 9 (17.3%); catheterization data n = 1 (1.9%) and non-failing Fontan group AST n = 45 (30.8%); ALT n = 42 (28.8%); bilirubin n = 63 (43.1%); NT-proBNP, eGFR n = 40 (27.4%); gGT, RDW n = 44 (30.1%); VO2peak n = 30 (20.6%); catheterization data n = 38 (26.0%); echocardiographic data n = 6 (4.1%)*.

*ACE, angiotensin converting enzyme; ALT, alanine aminotransferase; APC, atriopulmonary connection; AST, aspartate aminotransferase; AT1, angiotensin II type 1 receptor; AVC, atrioventricular connection; AVSD, atrioventricular septal defect; ccTGA, congenitally corrected TGA; CPET, cardiopulmonary exercise testing; eGFR, estimated glomerular filtration rate; gGT, γ-glutamyltransferase; HLHS, hypoplastic left heart syndrome; mPAP, mean pulmonary artery pressure; NT-proBNP, N-terminal pro-brain natriuretic peptide; PVRi, pulmonary vascular resistance index; RDW, red cell distribution width; SVEDP, systemic ventricular end-diastolic pressure; TCPC, total cavopulmonary connection; TCS, transcutaneous oxygen saturation; TGA, transposition of the great arteries; TPG, transpulmonary pressure gradient; VO_2_peak, peak oxygen uptake*.

### Failing Fontan Score

From the parameters collected, those with significant differences between patients with and without Fontan failure were considered to be included in the final score composition. The final score contained 15 items: ejection fraction, grade of atrioventricular valve incompetence, SVEDP, mPAP, PVRi, history of SVT, history of VT, presence of pacemaker, eGFR, RDW, NT-proBNP, gGT, transcutaneous oxygen saturation at rest (TCS), VO_2_peak and number of cardiovascular medications (excluding antithrombotic therapy). For the Fontan Failure Score, metric and ordinal variables were dichotomized. Cut-off values for dichotomization were pragmatically defined taking into account laboratory reference values, clinical considerations, previous definitions, distribution of values, discrimination between both groups by likelihood ratio and a minimal reduction of area under the curve (AUC) in receiver operating characteristic (ROC) curves by dichotomization. A score point was assigned for each value above the defined threshold and points summed up to the final Fontan Failure Score ranging from 0 to 15 points.

### Statistics

Statistical analyses were performed using SPSS (version 23, IBM Corp., Armonk, NY, USA) and R (version 4.0.2, R Foundation for Statistical Computing, Vienna, Austria), graphs were prepared using Prism 9.2 (GraphPad Software Inc., La Jolla, CA, USA). Data distribution was tested using D'Agostino-Pearson test. Variables are expressed as figures (percentages) and median (interquartile range, IQR). Continuous variables were compared using non-parametric Mann-Whitney test since the majority of variables displayed non-normal distribution. Fisher's exact test or Chi-Square-Test were used for categorical and ordinal data. Survival and freedom from transplantation or Fontan takedown as combined endpoint was assessed using Kaplan-Meier survival analysis. Receiver operating characteristic (ROC) curves were employed to assess accuracy of discriminating primary (Fontan failure) or secondary (mortality) outcomes. Potential risk factors for primary outcome were evaluated with univariable and multivariable logistic regression analysis. Those parameters with significant results (*p* < 0.05) in univariable analysis were considered for the multivariable model. Predictors were handled without transformation. To reduce the problem of overfitting and generation of invalid models caused by limited cohort sizes and number of events in combination with large numbers of analyzed predictors, the number of predictors entered into the multivariable model was limited to seven. Non-modifiable parameters (i.e., age at Fontan operation as well as the dichotomous categorical variables status post SVT, VT and pacemaker-implantation) and potentially modifiable parameters were analyzed separately. During model development, all combinations of 6–7 candidate predictors were entered and compared using the Akaike information criterion; patients with missing variables were excluded from analysis. In addition, a backward selection based on Akaike information criterion was performed. In all analyses, *p*-values <0.05 were considered statistically significant.

## Results

### Patient Cohort and Clinical Course of Fontan Failure

Patients' characteristics are summarized in Table 1. From a total of 573 Fontan patients followed in our institutions during the study period, 198 were adult at their last follow-up [median age 25.9 (IQR 21.4–32.8) years] and included in the study. Of them, 148 (74.7%) were originally operated in our institutions. From all adult Fontan patients, 52 (26.3%) were classified as patients with Fontan failure by our *a priori* defined criteria. Twenty-nine (55.8%) patients had heart failure corresponding to NYHA functional class III persisting for >12 months without clinical improvement, 18 (34.6%) had >2 unscheduled hospital admissions for worsening of heart failure symptoms within 12 months, 14 (26.9%) had active PLE and 8 (15.4%) patients had heart failure corresponding to NYHA functional class IV; 15 (28.8%) patients fulfilled more than one criterion of Fontan failure.

Of note, patients with Fontan failure were older at the time of Fontan procedure and at last follow-up (Table 1) while the median follow-up duration after Fontan procedure did not differ significantly compared to patients without failure. The most frequent underlying cardiac morphologies were tricuspid atresia and double inlet left ventricle and the majority had a left ventricular morphology of the systemic ventricle. Median follow-up in failing Fontan patients was 22.7 (IQR 15.2–25.7) years. Late clinical Fontan failure occurred at a median of 18.2 (IQR 9.1–21.1) years after Fontan completion. Freedom from Fontan failure in the entire cohort of adult patients was 91.7% at 10, 80.6% at 20, and 61.0% at 25 years after Fontan operation, respectively.

**Figure 1:**
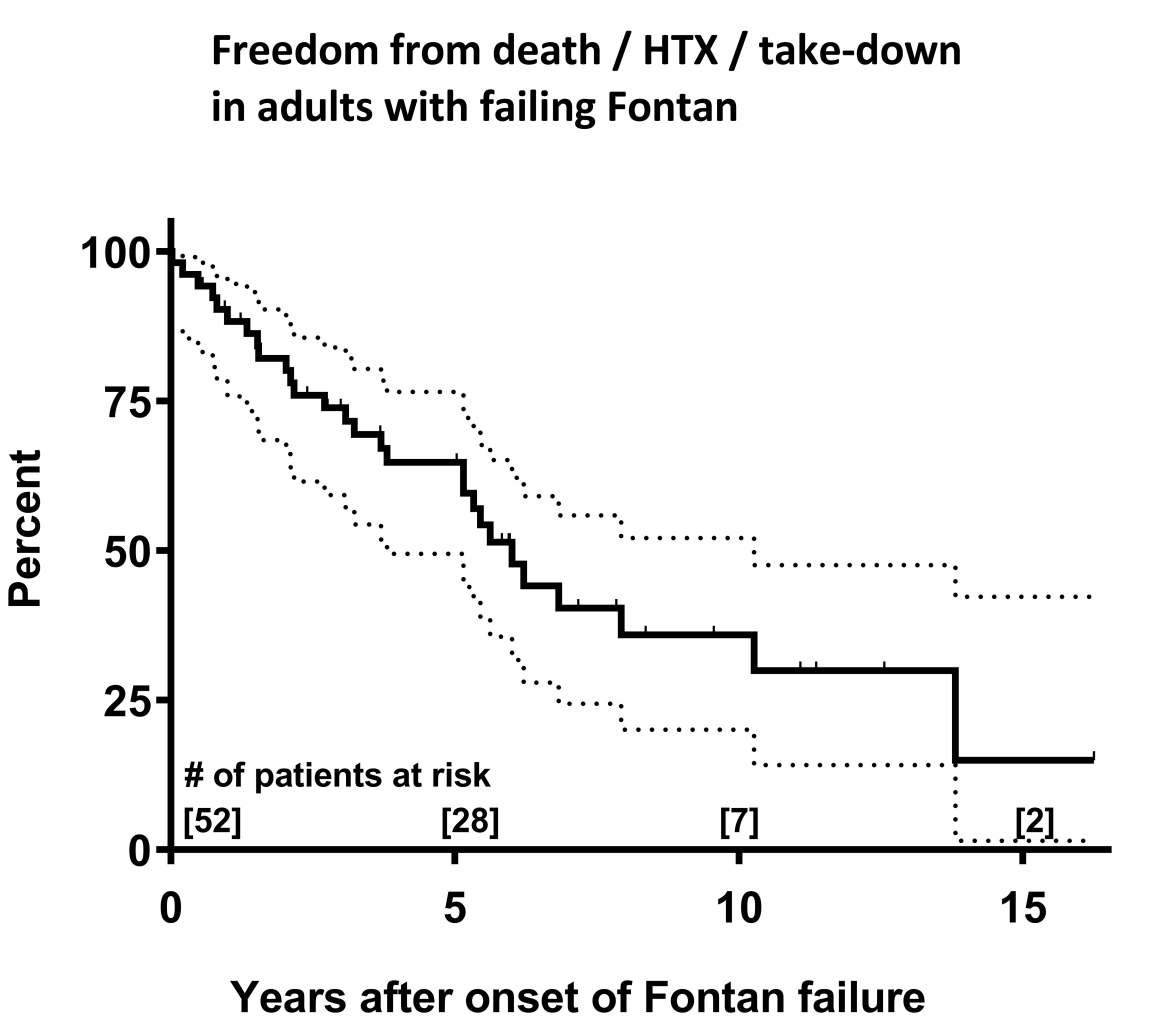
Kaplan-Meier survival estimates for the combined endpoint of survival and freedom from cardiac transplantation and Fontan take-down in failing Fontan patients after onset of failure. Dotted lines represent 95% confidence interval. HTX, cardiac transplantation.

Mortality of patients with Fontan failure was substantial, 25 (48.1%) patients died during follow-up. Kaplan-Meier estimates for freedom of death/transplantation/take-down were 64% at 5 years and 36% at 10 years after onset of Fontan failure, respectively (Figure 1). Causes of death were reoperation mortality (10/25, 40.0%), terminal circulatory failure/end-stage heart failure (5/25, 25.0%), sepsis/endocarditis with multiorgan failure (4/25, 16.0%), sudden cardiac death (3/25, 12.0%) and persistent heart failure despite mechanical circulatory support (3/25, 12.0%). In those who died, evaluation for cardiac transplantation was performed in 12/25 (48.0%) patients, of whom 4/12 (33.3%) were not accepted for transplantation by the institutional transplantation board due to precarious clinical condition and 1/12 (8.3%) patient declined cardiac transplantation. Four patients died on the waiting list and 2 patients during evaluation for transplantation. Overall, 5 patients with failing Fontan were transplanted with 2 early postoperative deaths due to graft failure requiring mechanical support and multiorgan failure, respectively.

### Variables Associated With Fontan Failure

Analyzed parameters are presented in Table 1 (graphed depiction in Supplementary Figure 2). Baseline characteristics in respect to underlying cardiac malformation, ventricular morphology and presence of heterotaxy did not differ significantly between patients with and without Fontan failure. However, extracardiac Fontan modification was more common in patients without failure compared to those with clinical Fontan failure. A large number of variables differed significantly between those patients with Fontan failure and those without failure in comparative analyses (Table 1) as well as univariable analyses (Table 2). Failing Fontan patients had higher Fontan/pulmonary artery and ventricular filling pressures, higher PVRi, reduced ejection fraction, higher degree of AVVI, lower oxygen saturation, reduced cardiopulmonary capacity, higher NT-proBNP values, impaired renal function and increased gGT. In addition, they were more likely to have a history of SVT or VT and pacemaker implantation and were administered a larger number of cardiovascular medications. As an interesting finding, RDW was also significantly higher in patients with Fontan failure. In the multivariable analyses, two models were developed analyzing potentially variable and non-modifiable predictors, respectively (Table 2). VO_2_peak, NT-pro-BNP, RDW and number of cardiovascular medications (multivariable model 1) as well as age at Fontan procedure, APC/AVC types of Fontan modification, presence of pacemaker and history of VT (multivariable model 2) were independent predictors of Fontan failure.

**Table 2:** Univariable and multivariable analysis for Fontan failure.

**Univariable analysis**				
**Variable**	**Odds ratio**	**95% CI**	***p***	***n***
Age at Fontan (years)	1.075	1.033; 1.119	**<0.001**	198
Age at last follow-up (years)	1.078	1.038; 1.118	**<0.001**	198
Ventricular morphology LV (*n*)	0.722	0.334; 1.563	0.401	198
Heterotaxy (*n*)	0.853	0.265; 2.742	0.789	198
Fontan modification APC/AVC (*n*)	8.059	3.330; 19.505	**<0.001**	198
TCS (%)	0.781	0.711; 0.858	**<0.001**	190
Ejection fraction (%)	0.920	0.886; 0.955	**<0.001**	194
AVVI >moderate (*n*)	5.368	2.589; 11.131	**<0.001**	190
mPAP (mmHg)	1.360	1.203; 1.537	**<0.001**	155
SVEDP (mmHg)	1.215	1.099; 1.342	**<0.001**	151
TPG (mmHg)	1.097	0.848; 1.420	0.481	153
PVRi (WU*m^2^)	2.215	1.273; 3.854	**0.005**	143
RDW (%)	2.138	1.694; 2.697	**<0.001**	180
eGFR (mL/min/1.73 m^2^)	0.964	0.948; 0.981	**<0.001**	156
AST/GOT (U/L)	1.005	0.989; 1.021	0.403	155
ALT/GPT (U/L)	0.992	0.978; 1.005	0.226	166
gGT (U/L)	1.004	1.000; 1.007	**0.035**	169
Total bilirubin (mg/dL)	1.296	0.948; 1.774	0.104	147
NT-proBNP (pg/mL)	1.001	1.001; 1.002	**<0.001**	166
CPET VO_2_peak (% of reference)	0.854	0.814; 0.897	**<0.001**	174
Pacemaker (*n*)	4.081	2.055; 8.103	**<0.001**	198
s/p SVT (*n*)	4.889	2.493; 9.589	**<0.001**	198
s/p VT (*n*)	9.524	2.880; 31.495	**<0.001**	198
Cardiovascular medications (*n*)	4.373	2.893; 6.608	**<0.001**	198
**Multivariable analysis (model 1, potentially variable predictors)**				***n =*** **124**
RDW (%)	3.566	1.111; 11.440	**0.033**	
NT-proBNP (pg/mL)	1.005	1.000; 1.011	**0.047**	
CPET VO_2_peak (% of reference)	0.825	0.684; 0.994	**0.043**	
Ejection fraction (%)	1.032	0.860; 1.239	0.743	
Cardiovascular medications (*n*)	6.188	1.135; 33.731	**0.035**	
**Multivariable analysis (model 2, non-modifiable predictors)**				***n =*** **198**
Age at Fontan (years)	1.058	1.012; 1.105	**0.012**	
Fontan modification APC/AVC (*n*)	4.193	1.502; 11.701	**0.006**	
Pacemaker (*n*)	2.497	1.116; 5.588	**0.026**	
s/p SVT (*n*)	2.204	0.965; 5.035	0.061	
s/p VT (*n*)	7.811	2.030; 30.051	**0.003**	

*Parameters with units in parentheses and number of cardiovascular medications were entered as continuous variables; the remaining parameters were entered as categorical variables. Degree of atrioventricular valve regurgitation as well as type of Fontan modification were dichotomized for analysis as >mild vs. none/mild and APC/AVC vs. extracardiac and intracardiac TCPC, respectively. VO_2_peak from cardiopulmonary exercise testing was analyzed as percentage of patient's age and sex-specific reference values*.

*P-values < 0.05 are considered statistically significant and highlighted in bold*.

*ALT, alanine aminotransferase; APC, atriopulmonary connection; AST, aspartate aminotransferase; AVC, atrioventricular connection; CI, confidence interval; CPET, cardiopulmonary exercise testing; eGFR, estimated glomerular filtration rate; gGT, γ-glutamyltransferase; mPAP, mean pulmonary artery pressure; NT-proBNP, N-terminal pro-brain natriuretic peptide; PVRi, pulmonary vascular resistance index; RDW, red cell distribution width; SVEDP, systemic ventricular end-diastolic pressure; SVT, supraventricular tachycardia; TCS, transcutaneous oxygen saturation; TCPC, total cavopulmonary connection; TPG, transpulmonary pressure gradient; VO_2_peak, peak oxygen uptake; VT, ventricular tachycardia*.

### Composition and Accuracy of Fontan Failure Score

Our final Fontan Failure Score includes a set of 15 clinical, echocardiographic, invasive hemodynamic and laboratory parameters: EF, grade of AVVI, SVEDP, mPAP, PVRi, eGFR, NT-proBNP, gGT, RDW, percentage of reference VO_2_peak, TCS, history of SVT, history of VT, presence of pacemaker and number of cardiovascular medications. Non-dichotomous variables were dichotomized as follows: EF ≤ 45%, AVVI > mild, SVEDP ≥ 12 mmHg, mPAP ≥15 mmHg, PVRi ≥2.5 WU^*^m^2^, eGFR < 90 ml/min/1.73 m^2^, RDW > 14.5%, NT-pro-BNP > 500 pg/mL, gGT > 100U/L, TCS at rest < 93%, VO_2_peak < 50% of reference and >2 cardiovascular medications. For each score item value beyond the defined threshold, one score point was assigned and points summed up to the final Fontan Failure Score ranging from 0 to 15 points.

**Figure 2:**
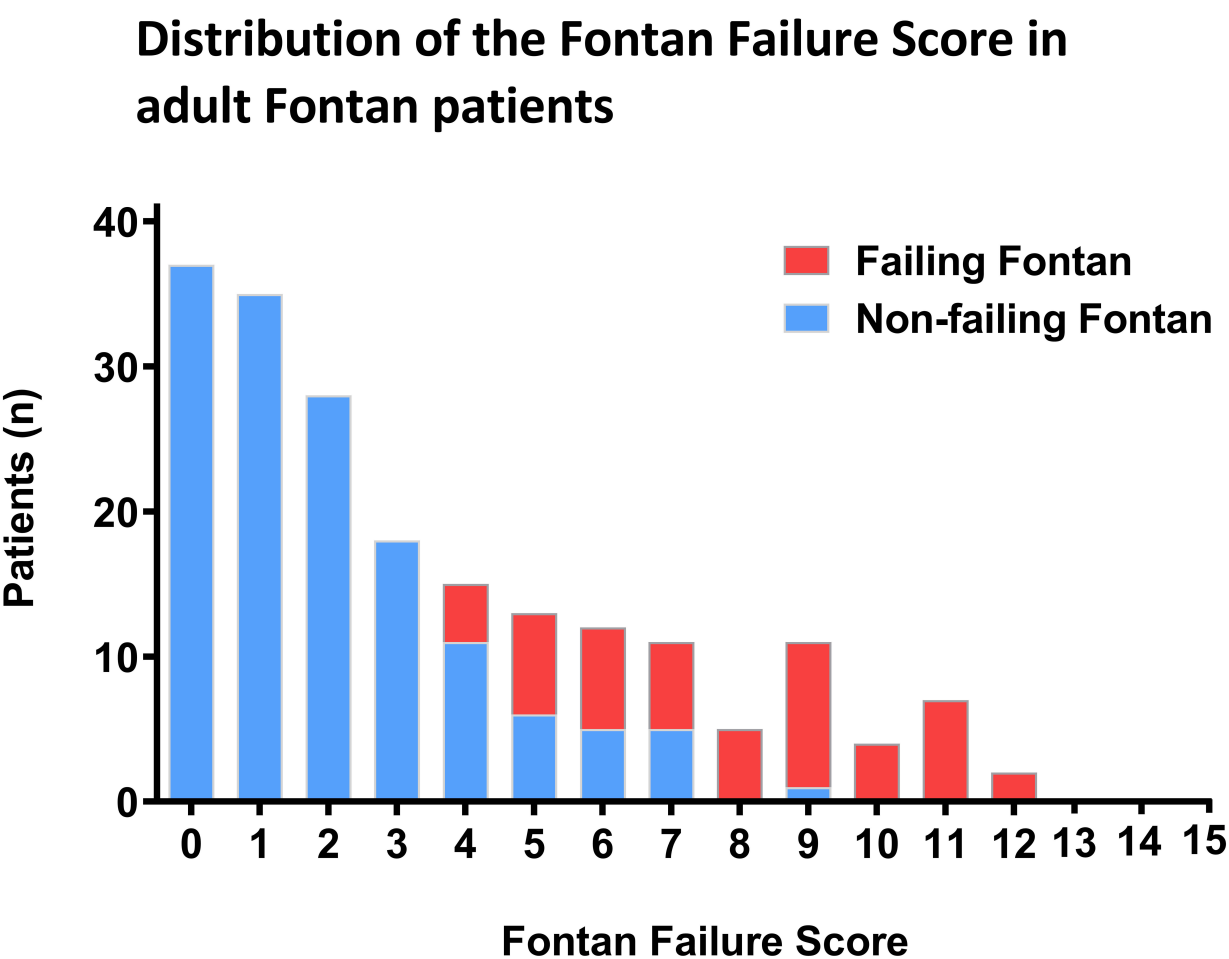
Distribution of the Fontan Failure Score in our cohort of adult patients. Columns represent number of patients stratified for those with (red) and without (blue) Fontan failure. The abscissa indicates summed points of the Fontan Failure Score.

**Figure 3:**
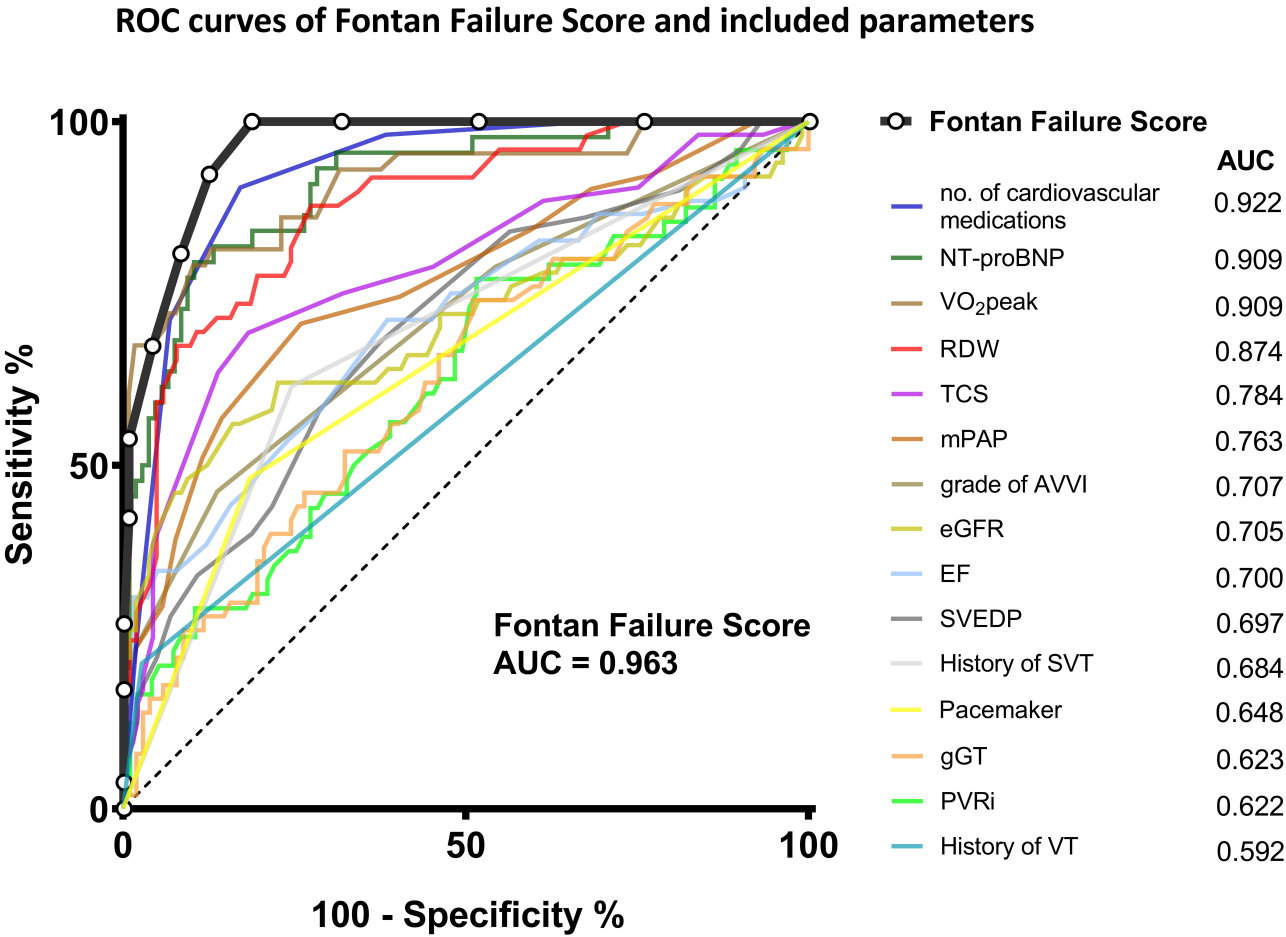
Receiver operating characteristic curves of the Fontan Failure Score and its components to discriminate Fontan failure in our cohort of adult Fontan patients. Area under the curve for each parameter is indicated in the legend. AUC, area under the curve; AVVI, atrioventricular valve incompetence; EF, ejection fraction; eGFR, estimated glomerular filtration rate; gGT, γ-glutamyl transferase; mPAP, mean pulmonary artery/Fontan pressure; NT-proBNP, N-terminal pro-brain natriuretic peptide; PVRi, pulmonary vascular resistance index; RDW, red cell distribution width; ROC, Receiver operating characteristic; SVEDP, single ventricular end-diastolic pressure; SVT, supraventricular tachycardia; TCS, transcutaneous oxygen saturation at rest; VO2peak, peak oxygen uptake in cardiopulmonary exercise testing; VT, ventricular tachycardia.

The distribution of the Fontan Failure Score values stratified for patients with and without Fontan failure is depicted in Figure 2. Patients with Fontan failure had a significantly higher median Fontan Failure Score compared to non-failing Fontan patients [8 points (IQR 5–10) vs. 2 point (IQR 1–5), *p* < 0.001]. The score showed a remarkable accuracy in discriminating Fontan failure patients as assessed by ROC analysis. Area under the curve (AUC) of the Fontan failure score was 0.963 (95% CI 0.921; 0.985, *p* < 0.001) to discriminate failure (Figure 3). With a cut-off of ≥4 points in the Fontan Failure Score, sensitivity was 100.0% and specificity 81.4% with a positive predictive value (PPV) of 65.0% and a negative predictive value (NPV) of 100.0% to classify Fontan failure (Table 3). In univariable analysis, the Fontan Failure Score was a significant predictor of Fontan failure (*p* < 0.001) with an odds ratio of 2.647 (95% CI 1.981; 3.536) per score point. Also mortality was accurately classified by the Fontan Failure Score with an AUC of 0.916 (95% CI 0.873; 0.959, *p* < 0.001). For the discrimination of mortality, sensitivity was 100.0%, specificity 69.0%, PPV 33.8%, NPV 100.0% for a threshold of ≥4 points of the Fontan Failure Score (Table 3). Median Fontan Failure Score in patients that died during follow-up was significantly higher compared to survivors [9 points (IQR 6–11) vs. 2 points (IQR 1–4), *p* < 0.001].

**Table 3:** Fontan Failure Score accuracy according to thresholds.

**Threshold Fontan failure score**	**Sensitivity (%) (95% CI)**	**Specificity (%) (95% CI)**	**PPV (%)**	**NPV (%)**	**+LR**	**–LR**
**Primary outcome: Fontan failure**						
4	100.0 (93.2; 100.0)	81.4 (74.1; 87.4)	65.0	100.0	5.2	0.0
5	92.3 (81.5; 97.7)	87.6 (81.1; 92.5)	73.8	97.0	7.9	0.1
6	80.8 (67.5; 90.4)	91.7 (86.0; 95.7)	78.8	92.5	10.5	0.2
7	67.3 (52.9; 79.7)	95.7 (91.2; 98.5)	85.0	88.6	15.9	0.4
8	53.9 (39.5; 67.8)	99.3 (96.2; 99.9)	96.6	85.8	78.6	0.5
9	42.3 (28.7; 56.8)	99.3 (96.2; 99.9)	95.8	83.3	64.6	0.6
10	26.9 (15.6; 41.0)	100.0 (97.5; 100)	100.0	78.9	-	0.8
11	17.3 (8.2; 30.3)	100.0 (97.5; 100)	100.0	77.2	-	0.8
12	3.9 (0.5; 13.2)	100.0 (97.5; 100)	100.0	74.5	-	1.0
**Secondary outcome: mortality**						
4	100.0 (87.2; 100.0)	69.0 (61.5; 75.8)	33.8	100.0	3.2	0.0
5	92.6 (75.7; 99.1)	76.6 (69.5; 82.7)	38.5	98.5	4.0	0.1
6	77.8 (57.7; 91.4)	81.9 (75.3; 87.3)	40.4	95.9	4.3	0.3
7	74.1 (53.7; 88.9)	88.3 (82.5; 92.7)	50.0	95.6	6.3	0.3
8	63.0 (42.4; 80.6)	93.0 (88.1; 96.3)	58.6	94.1	9.0	0.4
9	51.9 (32.0; 71.3)	94.2 (89.5; 97.2)	58.3	92.5	8.9	0.5
10	33.3 (16.5; 54.0)	97.7 (94.1; 99.4)	69.2	90.3	14.3	0.7
11	25.9 (11.1; 46.3)	98.8 (95.8; 99.9)	77.8	89.4	22.2	0.7
12	7.4 (0.9; 24.3)	100.0 (97.9; 100.0)	100	87.2	-	0.9

*+LR, positive likelihood ratio; –LR, negative likelihood ratio; CI, confidence interval; NPV, negative predictive value; PPV, positive predictive value*.

## Discussion

In our study, we have developed an uncomplex yet remarkably accurate score to classify Fontan failure in a comparably large cohort of adult Fontan patients. Our multimodal Fontan Failure Score is composed of several clinical, hemodynamic, echocardiographic and laboratory variables usually collected during routine follow-up examinations of Fontan patients which makes it a feasible means to systematically assess Fontan patients for signs of failure and may provide an estimate of severity of Fontan failure.

Acknowledging the truly unique success achieved by the Fontan palliation in allowing the survival of patients with formerly virtually lethal cardiac malformations into adulthood, the experiences from the past decades have led to the sobering recognition that the Fontan procedure is nevertheless of a palliative character only (25). The hemodynamic characteristics of the Fontan circulation are profoundly unphysiological and the increasing availability of long-term follow-up data in a growing number of patients in recent decades has demonstrated its significant intrinsic limitations (4, 8, 25, 26). In the absence of a subpulmonary ventricle in Fontan circulation, systemic ventricular preload and consequently cardiac output are critically dependent on passive pulmonary blood flow, which in turn is essentially dependent on a low pulmonary vascular resistance as well as preserved systolic and diastolic ventricular function. Unavoidably, the Fontan circulation pathophysiology results in chronic venous congestion and reduced ventricular preload with a chronic low output state (5, 27). As Fontan patients age, there is a continuous hemodynamic attrition of the Fontan circulation resulting in progressive heart failure and secondary organ dysfunctions with associated co-morbidities such as arrhythmias, exercise intolerance, cyanosis, protein-losing enteropathy, plastic bronchitis, Fontan associated liver disease, chronic kidney disease, frailty and coagulation disorders (7, 17, 27–31). The hemodynamic deterioration may have variable characteristics and courses but is considered to be ultimately inevitable, resulting in irreversible Fontan failure and premature death or the requirement of cardiac transplantation. As pointed out by recent population-based projections, the number of Fontan patients and consequently the number of these patients aging into adulthood will grow considerably (10). Accordingly, a substantial increase in the incidence of late Fontan failure and the number of adult Fontan patients experiencing severe and eventually life-expectancy limiting complications has to be expected within the near future.

### Fontan Failure Definition

Despite the general agreement that the Fontan circulation is predestinated to eventual failure, a common definition of Fontan failure was not available until recently, when consensus definitions of Fontan associated morbidities including “Fontan circulatory failure” have been presented (18). As much as these important efforts to systematize and harmonize scientific and clinical reporting have to be sincerely acknowledged, Fontan failure was, however, defined rather descriptively and imprecise, rendering the definition to be of limited practicability to exactly classify patients in care and in clinical research. The facts that signs and symptoms of Fontan failure are variable, often develop subtly over longer periods of time and circulatory decompensation may not necessarily be irreversible likely contribute to the difficulties in establishing an unambiguous definition. Moreover, it has been recognized that the assessment of heart failure in patients with congenital heart disease is challenging due to the patient's adaptability to their chronically reduced output state and often clinically unapparent deterioration (26, 32). In the present study, we have employed stringent, pragmatic clinical criteria to define Fontan failure in our cohort. These are arbitrary, however, we intended to encompass the variable spectrum of Fontan failure manifestation; not only including persistent heart failure, refractory protein-losing enteropathy and plastic bronchitis but also conditions with frequent circulatory decompensations requiring hospital admittance and therapeutic interventions such as recurrent dysrhythmias. Nonetheless, classification by our Fontan failure definition, is also certainly not unambiguous and might be prone to inconsistencies based on variabilities in patient assessment and institutional practices. Of note, there is accumulating evidence that Fontan failure is not a uniform process since the hemodynamic alterations observed in patients with failing Fontan may vary considerably (5, 6, 11, 12). This has resulted in the conceptualization of distinct hemodynamic phenotypes of Fontan failure (11). However, up to now, the proposed classifications of Fontan failure phenotypes are largely grounded on theoretical and observational considerations and await substantiation by systematic analysis of hemodynamic data in adequately sized patient cohorts. Future studies will have to invest further effort in the important task to more precisely characterize and define Fontan failure.

### Assessment of Fontan Failure

A large variety of parameters has been shown to be significantly associated with adverse late outcome after Fontan operation including anatomic features, peri- and postoperative variables, single ventricular function, atrioventricular valve regurgitation, arrhythmias, cardiopulmonary capacity and signs of secondary organ dysfunction among others (8, 9, 33–39). While these might be useful for a vague estimate of a given patients risk for adverse events, they are unfeasible for the assessment of the severity of hemodynamic impairments. Moreover, findings were not always consistent across studies, resulting in uncertainties concerning the appraisal of individual risk factors' impact on prognosis.

A clinical scoring system aiming at classifying Fontan failure and grading Fontan failure severity might prove valuable to address several urgent issues. It may allow a more reliable longitudinal evaluation of individual Fontan patients' clinical status and facilitate detecting deterioration of the Fontan circulation. Thereby it could help in determining the appropriate time points for therapeutic interventions and ultimately cardiac transplantation. In addition, by providing a more objective assessment of Fontan failure, it would allow more accurate interindividual comparisons in patients with heterogeneous phenotypes and severities of Fontan failure which would represent an important tool for future studies investigating outcome and interventions in patients with failing Fontan. Previously, a score to assess the risk of late mortality after Fontan operation has been proposed (40). The meta-analysis integrates data from various prior studies and includes a large number of Fontan patients. Such score might prove feasible in identifying Fontan patients with increased late mortality risk, however, the score is unable to identify Fontan failure or grade Fontan failure severity which would clearly be of broader clinical value since it would offer the possibility of timely interventions in order to prevent late mortality rather than assessing an overall mortality risk for an unknown future time frame. Moreover, the practicability of this score seems limited as it included several non-modifiable parameters such as preoperative hemodynamics, anatomic features and type of Fontan modification and several distinct risk factors have been combined to single score items (40). Therefore, we conducted the present study seeking to develop a practicable, uncomplex scoring system that yet accurately identifies patients with Fontan failure.

### Differences Between Failing and Non-failing Fontan Patients

In our cohort of adult Fontan patients we have assessed a variety of variables. While for most of the hemodynamic parameters studied, we observed quite anticipated results in failing Fontan patients in respect to overall reduced systolic ventricular function, increased ventricular filling pressures and Fontan pressures as well as slightly increased PVR (Table 1; Supplementary Figure 2), several of our additional findings are particularly notable.

For NT-proBNP or BNP as biomarkers for heart failure, previous studies have yielded somewhat inconsistent results in Fontan patients. One study reports a correlation with functional class of Fontan patients while others did not find a clear association with Fontan failure; overall, the number of studies on this subjects are quite limited (41–43). Moreover it has been reported that atriopulmonary Fontan modifications (APC) have higher BNP levels compared to extracardiac Fontan modifications (41, 42). In our cohort, we found a significant difference of NT-proBNP levels between failing and non-failing Fontan patients and therefore included it in our Fontan Failure Score. Analyzing different Fontan modifications, we also found significantly higher NT-proBNP levels in APC compared to the extracardiac modification in non-failing Fontan patients [483.8 (IQR 270.0; 787.5) vs. 78.4 (IQR 44.9; 172.2) pg/mL, *p* < 0.001, Supplementary Figure 3], however there was no significant difference between modifications in failing Fontan patients (*p* = 0.790, Supplementary Figure 3). We would conclude that although individual values and particular cut-offs might not accurately identify suboptimal Fontan hemodynamics, NT-proBNP nevertheless represents a useful parameter that could well indicate circulatory deterioration in Fontan patients during longitudinal follow-up.

In regard to RDW, it has previously been recognized to be associated with heart failure and predictive for resulting mortality in adults with normal biventricular anatomy, irrespective of the presence of anemia (44, 45). There is, to the best of our knowledge, only one previous report that studied this parameter in Fontan patients (46). The authors observed a correlation of increased RDW with impairment of Fontan hemodynamics in terms of increased central venous pressure and decreased mixed venous oxygen saturation and cardiac index. However, the study cohort was rather small-sized and included only children. Our study is the first to show that RDW is also a promising variable to indicate hemodynamic impairment in a large cohort of adult Fontan patients. RDW was significantly increased in adults with Fontan failure (Table 1) and it displayed a good discrimination between failing and non-failing Fontan patients (Figure 3). Since RDW is an inexpensive and readily available laboratory parameter, our data suggests that it should be included in the routine follow-up blood tests of Fontan patients.

Previously it has been reported that simple clinical variables such as oxygen saturation (TCS) are predictive for adverse outcome in adult Fontan patients (39). We also recently showed that cyanosis is an independent risk factor for mortality during long-term follow-up across age groups in Fontan patients (47). In our cohort, TCS is slightly but significantly lower in failing Fontan patients (Table 1) and satisfyingly classifies Fontan failure (Figure 3). Although reasons for cyanosis in Fontan patients are multifactorial, TCS is an appealingly simple parameter for the longitudinal assessment, indicative of attrition of the Fontan circulation. The same holds true for the number of cardiovascular medications. In previous studies, diuretic therapy has been related to late mortality after Fontan, likely representing a surrogate parameter for circulatory failure (36, 48). Not surprisingly, in our cohort the number of prescribed cardiovascular medications was significantly higher in failing Fontan patients, however, it unexpectedly accurately discriminated failing Fontan patients with an AUC of 0.922 (Figure 3). These simple variables collected during detailed standardized follow-up examinations by themselves may already have a good capacity to indicate deterioration and anticipate Fontan failure.

### Fontan Failure Score

Our final multimodal Fontan Failure Score is composed of a total of 15 parameters. These include well-established risk factors of adverse late outcome but also less recognized indicators, as discussed above. Choice of variables was also guided by the rationale to analyze parameters indicative of hemodynamic deterioration and secondary organ dysfunction commonly collected during comprehensive follow-up according to current recommendations (16). Also weighing up feasibility of the score by limiting components to a reasonable number of parameters that are routinely collected during follow-up against a maximum of discrimination accuracy as well as limiting the number of non-variable parameters which might reduce the score's ability to indicate clinical improvement during longitudinal assessments were taken into consideration during score composition.

Our score, however, was only evaluated in our development cohort and yet requires external validation to reliably assess its diagnostic precision and validity with a priori set cut-offs. It will also likely need refinement and calibration. Moreover, our Fontan Failure Score offers the possibility of grading Fontan failure severity, however to assess the appropriateness of grading and determining meaningful discriminating cut-offs, longitudinal studies will be required. Various additional diagnostic modalities and parameters that may potentially improve accuracy of the Fontan Failure Score were not investigated in our study. In agreement with previous studies, laboratory parameters indicative of hepatic injury such as transaminases and bilirubin were in general only marginally elevated in the Failing Fontan patients although Fontan-associated liver disease (FALD) is a frequent co-morbidity during long-term follow-up (31, 49, 50). Only gGT showed a significant increase in our patients with Fontan failure and was included in the Fontan Failure Score. Nonetheless, also gGT is reported to only inconsistently correlate with severity of chronic hepatic injury in FALD (49, 50). Other non-invasive modalities assessing the extent of FALD that can easily be implemented in routine follow-up examinations such as hepatic ultrasound and transient elastography might be superior indicators of FALD progression and thereby deterioration of Fontan hemodynamics. Concerning CPET, reduced cardiopulmonary capacity indicating limited cardiac output as a result of compromised Fontan hemodynamics is a known predictor of adverse late events and mortality (37, 51). Nevertheless, additional parameters assessed in CPET beyond VO_2_peak such as limited heart rate reserve have been shown to correlate with adverse outcome and might classify Fontan failure more accurately (48, 52). Moreover, additional laboratory parameters such as serum uric acid, previously observed to be related to reduced exercise capacity and unfavorable outcome in adult Fontan patients, might prove valuable to increase our score's precision to classify Fontan failure (53).

In addition, several components of our score may be affected by confounders. For example, number of cardiovascular medication is likely influenced by practice variability among institutions, which has been reported to be considerable; a fact that is largely based on the profound lack of evidence concerning heart failure treatment in failing Fontan patients (54, 55). Also increased PVR, as an important pathophysiologic component of Fontan failure, will be affected by pulmonary vasodilator treatment. In fact, more than half of our failing Fontan patients received pulmonary vasodilators. In respect to renal function assessment, it has been suggested that creatinine-based determinations of eGFR overestimate actual glomerular filtration rate and cystatin C-based eGFR might be more reliable (30, 56, 57). In our study, cystatin C was available in only 16 (33.3%) of the failing Fontan patients and in 56/198 (28.3%) of the entire cohort of adult Fontan patients. Nonetheless, comparing both methods of eGFR determination in our cohort showed higher eGFR values in the creatinine-based calculation with a fixed bias (mean 17.9 ± 12.7 ml/min/1.73 m^2^, corresponding to 18.6 ± 14.2%, *p* < 0.001) as assessed by Bland-Altman plot. Thus, the method of eGFR calculation likely affects the assessment of renal function and thereby potentially the Fontan Failure Score.

Yet, despite these restrictions and potential confounders, our Fontan Failure Score shows a remarkable diagnostic accuracy, precisely discriminating patients with and without Fontan failure in our cohort with an AUC of 0.963 in ROC analysis. In addition, also mortality was accurately classified by the Fontan Failure Score. These promising results suggest that our proposed Fontan Failure Score may represent a useful and precise clinical tool to identify patients with developing Fontan circulation failure that may facilitate diagnostic and therapeutic decision making in the long-time care of Fontan patients. It therefore represents an important step towards a more accurate identification and systematic assessment of late Fontan failure.

### Limitations

Beyond limitations already discussed, additional limitations of this study are inherent to its restriction to patients from two institutions as well as its retrospective observational design for which consistency in data acquisition and follow-up examinations are not given. Not all parameters were available for the entire cohort, which potentially introduces some selection bias and, importantly, limits statistical analyses and multivariable modeling. Thresholds of dichotomized parameters were set reasonably according to distribution and discrimination; however, they are nonetheless arbitrary. Interpretability of peak VO_2_, as effort-dependent variable, is limited since we did not record parameters indicating exercise effort. Fontan failure criteria were retrospectively determined from medical records but clinical symptoms were not assessed which might result in some misclassification bias. We only analyzed patient's last follow-up visit and retrieved information from medical records to define the time point of fulfilling our criteria for Fontan failure. However, our analyses do not take longitudinal clinical changes of patients in respect to periods of improvement or decompensation between these time points and its relation to the proposed Fontan Failure Score into account. Therefore, future prospective longitudinal studies are indispensable to validate our score, evaluate its accuracy to predict Fontan failure and assess the impact of initiated therapies. Echocardiographic determination of single ventricular function has important limitations. More reliable modalities such as cardiac MRI were only inconsistently performed and therefore not analyzed. We have only assessed adult Fontan patients and our Fontan Failure Score might yield differing results in the pediatric age group, since Fontan failure in children is likely to be governed by distinct mechanisms (58, 59).

## Conclusions

We have developed an uncomplex but remarkably accurate score to classify Fontan failure and late mortality in adult Fontan patients. The Fontan Failure Score is comprised of several clinical, hemodynamic, echocardiographic and laboratory parameters usually collected during routine follow-up examinations. This renders the score a feasible means to assess Fontan patients for signs of failure. Prospective longitudinal validation and most likely refinement and calibration of the score in larger and preferably multi-institutional cohorts are required to explore its potential to grade Fontan failure severity and to actually predict the risk of Fontan failure and late mortality.

## Data Availability Statement

The raw data supporting the conclusions of this article will be made available by the authors, without undue reservation.

## Ethics Statement

The study involving human participants was reviewed and approved by Ethics Committee of the Charité - Universitätsmedizin Berlin. Written informed consent for participation was not required for this study in accordance with the national legislation and the institutional requirements.

## Author Contributions

PK conceptualized the study, performed data acquisition and analysis, and drafted the manuscript. SO and FB were involved in conceptualization and data analysis as well as review and editing. AS, MS, FD, JN, and FB helped in data acquisition and reviewed and edited the manuscript. All authors approved the final version of the manuscript.

## Funding

This study was supported by a contribution from the charitable foundation Stiftung KinderHerz for research dedicated to Fontan patients.

## Conflict of Interest

The authors declare that the research was conducted in the absence of any commercial or financial relationships that could be construed as a potential conflict of interest.

## Publisher's Note

All claims expressed in this article are solely those of the authors and do not necessarily represent those of their affiliated organizations, or those of the publisher, the editors and the reviewers. Any product that may be evaluated in this article, or claim that may be made by its manufacturer, is not guaranteed or endorsed by the publisher.
