# Supplementary material for: A Multimodal Score Accurately Classifies Fontan Failure and Late Mortality in Adult Fontan Patients
Source: Front Cardiovasc Med. 2022 Mar 10;9:767503. doi: 10.3389/fcvm.2022.767503 (PMC8960137; doi:10.3389/fcvm.2022.767503)
Supplement: Supplementary file 1 [file Image_1.pdf]

Study cohort flowchart

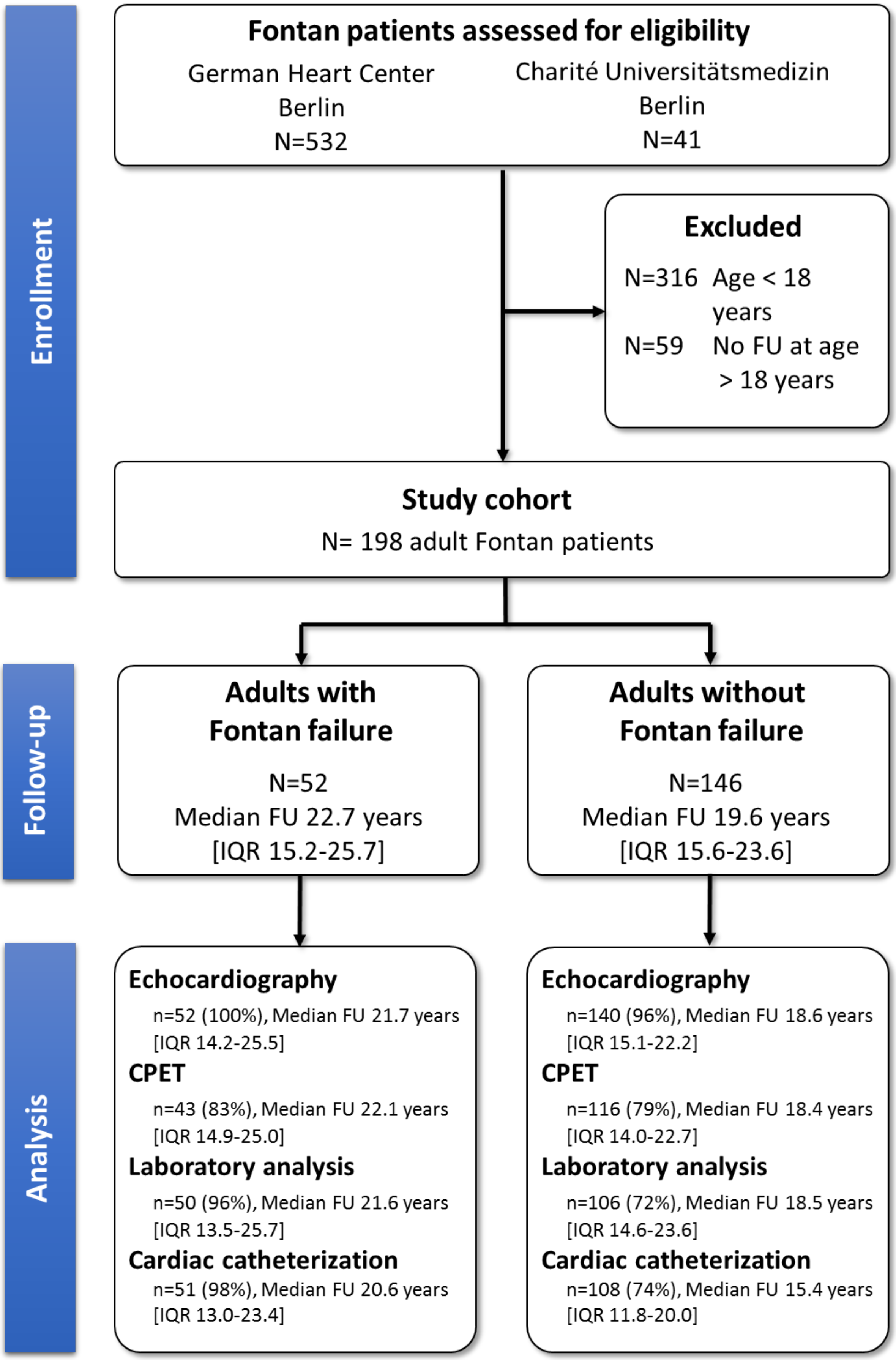

Supplemental Figure 1

Flowchart depicting the study cohort. Number of available examinations with parameters included in the study analysis are given with respective median follow-up duration after Fontan operation. Note that not all laboratory parameters were available from the last blood sample analyses in all patients (see also Table 1).

CPET - cardiopulmonary exercise testing; FU - follow-up; IQR - interquartile range.
