# Supplementary material for: A Multimodal Score Accurately Classifies Fontan Failure and Late Mortality in Adult Fontan Patients
Source: Front Cardiovasc Med. 2022 Mar 10;9:767503. doi: 10.3389/fcvm.2022.767503 (PMC8960137; doi:10.3389/fcvm.2022.767503)
Supplement: Supplementary file 2 [file Image_2.pdf]

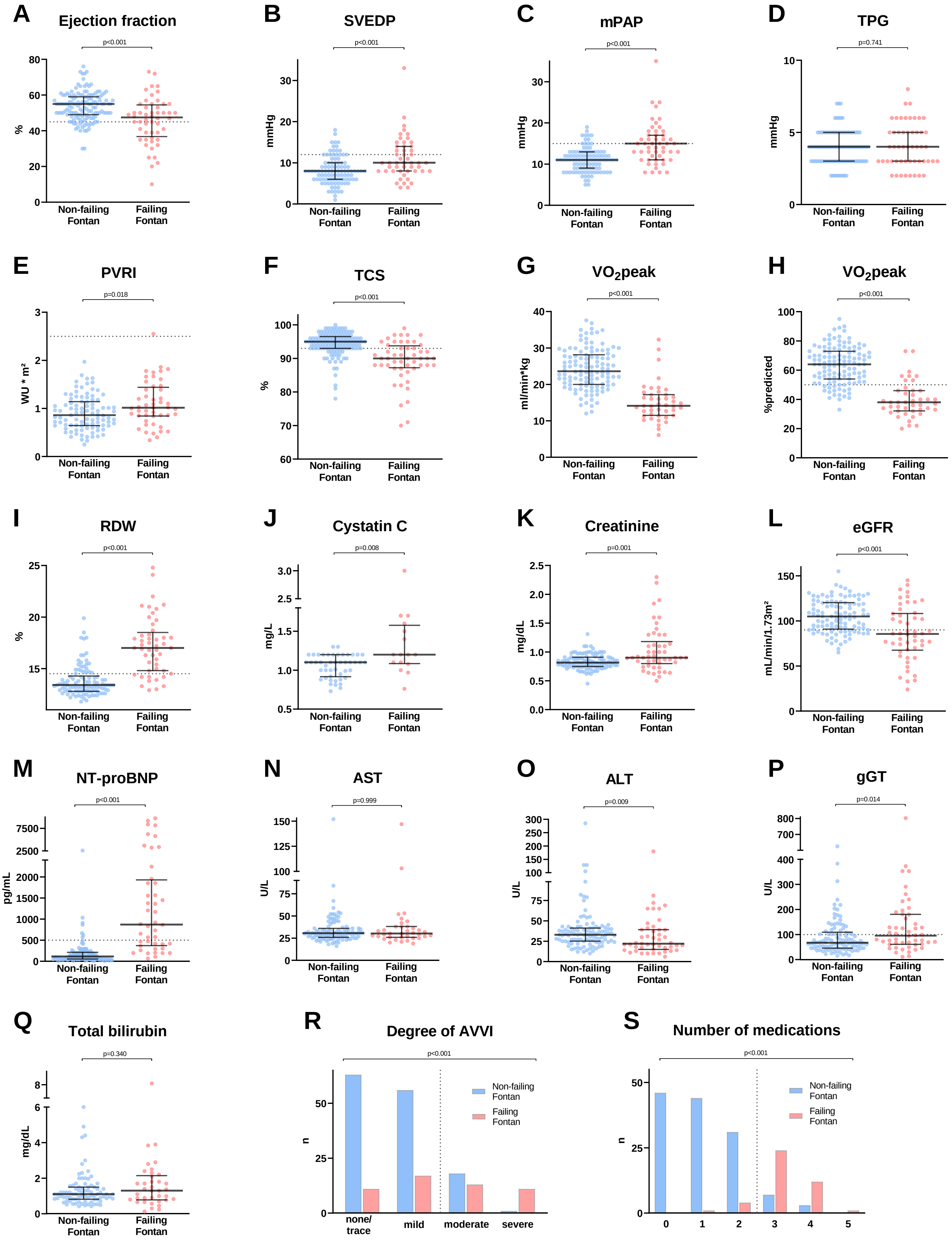

A
Ejection fraction
p<0.001
%
Non-failing Fontan
Failing Fontan
B
SVEDP
p<0.001
mmHg
Non-failing Fontan
Failing Fontan
C
mPAP
p<0.001
mmHg
Non-failing Fontan
Failing Fontan
D
TPG
p=0.741
mmHg
Non-failing Fontan
Failing Fontan
E
PVRI
p=0.018
WU * m²
Non-failing Fontan
Failing Fontan
F
TCS
p<0.001
%
Non-failing Fontan
Failing Fontan
G
VO2peak
p<0.001
ml/min*kg
Non-failing Fontan
Failing Fontan
H
VO2peak
p<0.001
%predicted
Non-failing Fontan
Failing Fontan
I
RDW
p<0.001
%
Non-failing Fontan
Failing Fontan
J
Cystatin C
p=0.008
mg/L
Non-failing Fontan
Failing Fontan
K
Creatinine
p=0.001
mg/dL
Non-failing Fontan
Failing Fontan
L
eGFR
p<0.001
mL/min/1.73m²
Non-failing Fontan
Failing Fontan
M
NT-proBNP
p<0.001
pg/mL
Non-failing Fontan
Failing Fontan
N
AST
p=0.999
U/L
Non-failing Fontan
Failing Fontan
O
ALT
p=0.009
U/L
Non-failing Fontan
Failing Fontan
P
gGT
p=0.014
U/L
Non-failing Fontan
Failing Fontan
Q
Total bilirubin
p=0.340
mg/dL
Non-failing Fontan
Failing Fontan
R
Degree of AVVI
p<0.001
n
Non-failing Fontan
Failing Fontan
none/ trace
mild
moderate
severe
S
Number of medications
p<0.001
n
Non-failing Fontan
Failing Fontan
0
1
2
3
4
5

Supplemental Figure 2

Comparison of variables in failing and non-failing adult Fontan patients (Table 1). Continuous variables (A-Q) are depicted as scatterplots, categorical variables (R-S) as column diagrams. In variables with large ranges, ordinates have been segmented for better visualization; segments were set to graph all data points. A-Q: Dots represent individual data points for non-failing (blue) and failing Fontan patients (red); central lines indicate median and interquartile range. R-S: columns represent frequency of grade of atrioventricular valve incompetence (R) and number of prescribed cardiovascular medications (S) for non-failing (blue) and failing Fontan patients (red). For variables included in the Fontan Failure Score, dichotomizing thresholds are indicated by dotted lines.

ALT - alanine aminotransferase; AST - aspartate aminotransferase; AVVI - atrioventricular valve incompetence; eGFR - estimated glomerular filtration rate; gGT - γ-glutamyl transferase; mPAP - mean pulmonary artery/ Fontan pressure; NT-proBNP - N-terminal pro-brain natriuretic peptide; PVRI - pulmonary vascular resistance index; RDW - red cell distribution width; SVEDP - single ventricular end-diastolic pressure; TCS - transcutaneous oxygen saturation at rest; VO2peak - peak oxygen uptake in cardiopulmonary exercise testing; WU - Wood units.
