# Supplementary material for: A Multimodal Score Accurately Classifies Fontan Failure and Late Mortality in Adult Fontan Patients
Source: Front Cardiovasc Med. 2022 Mar 10;9:767503. doi: 10.3389/fcvm.2022.767503 (PMC8960137; doi:10.3389/fcvm.2022.767503)
Supplement: Supplementary file 3 [file Image_3.pdf]

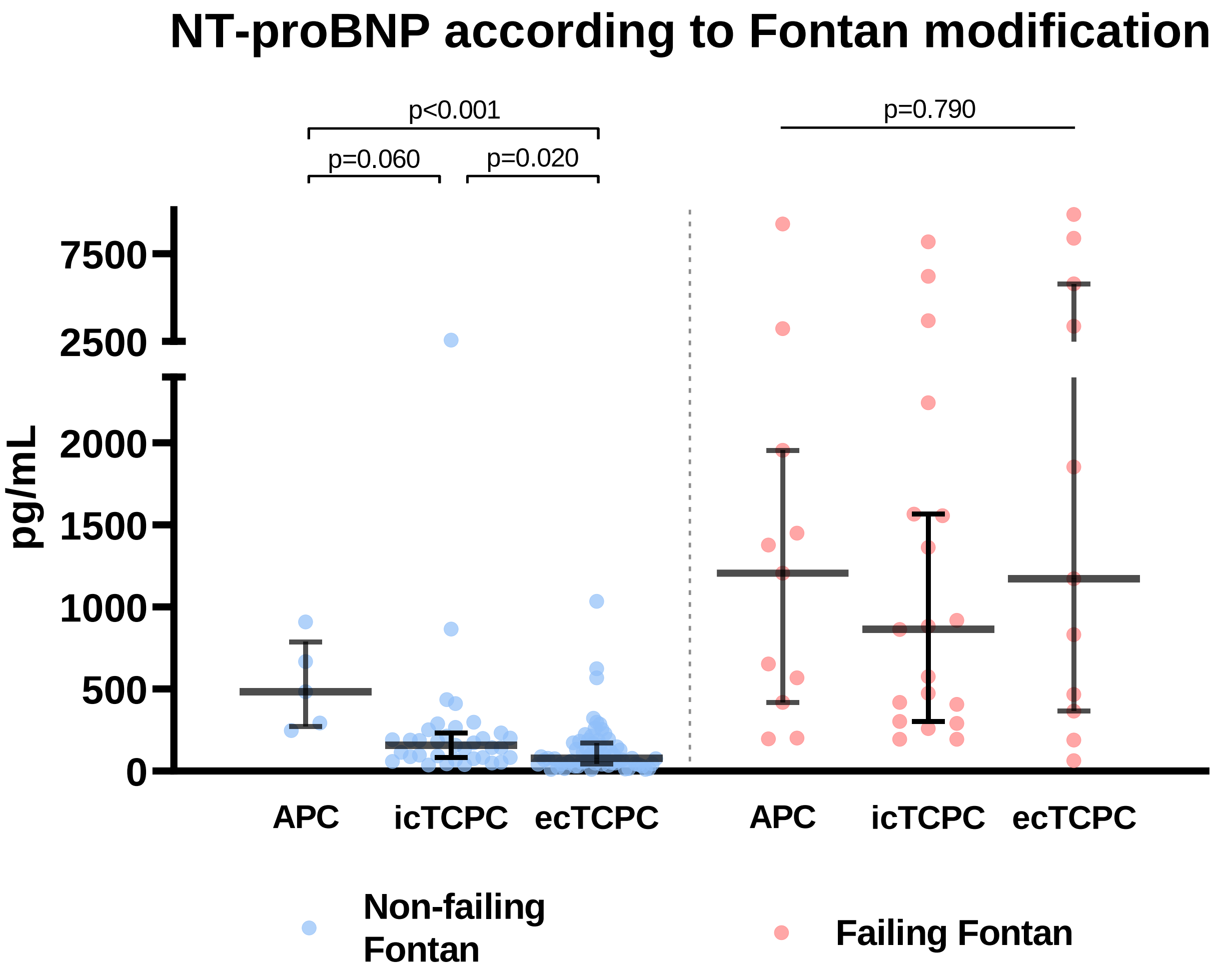

Supplemental Figure 3

Scatterplot of NT-proBNP levels in non-failing (blue) and failing Fontan patients (red) stratified for Fontan modifications. Central lines indicate median and interquartile range. Ordinate has been segmented for better visualization; segments were set to graph all data points. Data within non-failing / failing Fontan groups were compared by Kruskal-Wallis test with Dunn's multiple comparisons test.

APC- atriopulmonary connection; ecTCPC - extracardiac total cavopulmonary connection; icTCPC - intracardiac total cavopulmonary connection; NT-proBNP - N-terminal pro-brain natriuretic peptide.
